# Supplementary material for: Genome-wide identification of ABCC gene family and their expression analysis in pigment deposition of fiber in brown cotton (Gossypium hirsutum)
Source: PLoS One. 2021 May 7;16(5):e0246649. doi: 10.1371/journal.pone.0246649 (PMC8104370; doi:10.1371/journal.pone.0246649)
Supplement: S1 Table — (DOCX) [file pone.0246649.s001.docx]

**S1 Table. Sequences of primers used for RT-PCR in this study.**

| **Gene** | **Forward primer (5′-3′)** | **Reverse primer (5′-3′)** |
| --- | --- | --- |
| UBQ | GAAGGCATTCCACCTGACCAAC | CTTGACCTTCTTCTTCTTGTGCTTG |
| GhABCC1 | AGTGGTTGTCTCGGAATG | AGTGGTGTCTTGGGAAGT |
| GhABCC2 | AGGTAGGGAGAAGACTTTG | GCTGTGATGATGGGTTGG |
| GhABCC3 | TAAACGCTCACAATAACAC | CACGACCAAGAAGAACTA |
| GhABCC4 | AGTGGGACAATTCGTGAC | TCTTCGCTAGAGCAGTCA |
| GhABCC5 | TACGCTTTATCACTGACC | ACTCCTACTCTGTTCCCT |
| GhABCC6 | CCTACTGGCTGGCGTTTG | ATGGCTCCGATTGTTGCT |
| GhABCC7 | GAGGAGATGCGGGATGTA | GAGAGACCTTTGTTTGTGCC |
| GhABCC8 | GCTCCTATGTCTTTCTTCG | ATACGAGTCGGTCAGCAC |
| GhABCC9 | CAGCTAGATAGCCACGAT | ACTAAAGTCACCCACCCT |
| GhABCC10 | CACTGACCGACTCGTATT | ACTGCCTGTTCTCCCTA |
| GhABCC11 | ATGCCCAACAACTACAAC | GAATCAAGAATCCACCCT |
| GhABCC12 | GAATCAAGAATCCACCCT | CGCTATCAAGAACCGAAGT |
| GhABCC13 | ACGACTCAGCATCATCCCTC | GCCTCCCAAATCTGTTCATC |
| GhABCC14 | GTTCGTGCTCACCCTCCTA | TCCAAGGCTGCTGGCTACT |
| GhABCC15 | GCAAGAGCCCATCAGGTC | TGTATGCGGTCGTCCCAC |
| GhABCC16 | GGGGCTAAAGACATCACAA | GATCACTGGATACCCGACT |
| GhABCC17 | AAGCAAACCCAAAGTGAG | TAGGACCAACATACATAACG |
| GhABCC18 | TTCCATAACTCCAGCACA | CTTCGGTCTACAATCCTC |
| GhABCC19 | GCCTCGACTCAATCACTA | AAACGGAATCCCAACCAC |
| GhABCC20 | AGGCTTACGTGCTGACC | GAAACCAATACGGAAACTC |
| GhABCC21 | TGCAGGGCTTAATACAAT | GAGCACCGAAGTACCACA |
| GhABCC22 | TGTAAACGCTCACAATAAC | CACGACCAAGAAGAACTA |
| GhABCC23 | TGCCGTAGATGCCCAAAC | CTCCTCAAATGCCGTCCC |
| GhABCC24 | GAGTCACCGTCCTCCACCT | TTCCTCCTGATTACATCCTTC |
| GhABCC25 | AACGGAAGGAACGATTGA | TGTGAAGGACCTCCCAGA |
| GhABCC26 | ATGGCTGGCGATTATTGGT | CGAGGCGAGTTAGTTCACG |
| GhABCC27 | ATGGCTGGCATCTTCTCG | GCTGTCCACCATCCCTCT |
| GhABCC28 | AGCAGCACTTGGTTACGC | TTATCGCCCACAATGAAAA |
| GhABCC29 | CTTCAGAGGCTCCAGACA | TCCAAGTTTAATCGCACA |
| GhABCC30 | CAGAACAGGAAGTGGGAAAT | CCTTATGGTGCCTTGAAAC |
| GhABCC31 | AGAGCCACCGTCCTCCACCT | ACAATGCTCGGGCAAGACAC |
| GhABCC32 | TTGATTCTGGCAGGGTTC | TTTCCAAGCGAGTAAGGT |
| GhABCC33 | AGAGCCGCAGTCCTCCACCT | AACAATGCTCGGGCAAGACAC |
| GhABCC34 | TCGGTCCATACCTCATCG | GCCCTCTGCCTAAGTCCA |
| GhABCC35 | CAGGGTTCCGATGTTTAT | TTTCCTACCCACTTCACG |
| GhABCC36 | TATCGCTCCAATGCTCC | CGAATGCTGCCTCTAAAT |
| GhABCC37 | AGTATGCTGGATTTGCTGGTT | TGTTGAGGATGAATGGGAGA |
| GhABCC38 | CTGGTGTCGCTTCCTGAT | TTCCCACTTCCTGTCCTT |
| GhABCC39 | ACTCCTATTCTCGTCCAA | ATTTACCACTGCCTGTTT |
| GhABCC40 | ACTGTTAGCGTGTTTGGA | GGATAGGACTGGAGGAATA |
| GhABCC41 | AGAGGGACTGTCGCTTAT | GGCACTTAAAGGGTCATC |
| GhABCC42 | GATGAACAGATTTGGGAGGC | TGTATTAGGTTGTCGGTGGC |
