# Supplementary material for: Genome-wide identification of ABCC gene family and their expression analysis in pigment deposition of fiber in brown cotton (Gossypium hirsutum)
Source: PLoS One. 2021 May 7;16(5):e0246649. doi: 10.1371/journal.pone.0246649 (PMC8104370; doi:10.1371/journal.pone.0246649)
Supplement: Attachment — Submitted filename: Response to Reviewers.docx [file pone.0246649.s002.docx]

Dear Editors and Reviewers,

Thank you and the reviewers for your comments concerning our manuscript entitled “Genome-wide Identification and Expression Analysis of ABCC Gene Family in *Gossypium hirsutum*” (PONE-D-20-32250). These comments are all valuable and very helpful for revising and improving our manuscript. We have revised the manuscript carefully according to the reviewers’ comments, and have made some corrections marked in the revised manuscript. Response to reviewers is appended below. We hope that these modifications will be satisfactory for you and the reviewers.

We appreciate you very much for your efforts on our manuscript. It would also be of our great pleasure if our work is acceptable for publication in PLOS ONE.

We are looking forward to hearing from you soon.

Sincerely yours,

Jun-Shan Gao

The main corrections are marked in the paper and the responds to the reviewer’s comments are as follows:

Responds to the reviewer’s comments:

Reviewer #1:

Question 1: Recommendation to modify the title as “Genome-wide Identification of ABCC Gene Family and Their Expression Analysis in Pigment Deposition of Fiber in Brown-fiber cotton”.

Response: Thank you very much for your suggestion. The title has been revised properly.

Question 2: As we know that brown-fiber cotton is tetraploid cultivar (AADD), thus, it is better to number the *ABCC* genes like 1A, 1D, 2A, 2D, etc, for a more clear understanding of these genes. While, it is also fine to name the *ABCC* genes as current format *GhABCC1-42*.

Response: Thank you very much for your comment. It is really true as you suggested that it is better to number the *ABCC* genes like 1A, 1D, 2A, 2D, etc, for a more clear understanding of these genes. However, after comprehensive consideration, for the convenience of subsequent experiments, we decided to name the *ABCC* gene as the current format *GhABCC1-42*.

Question 3: All the legends of the figures are not sufficient to explain the figures.

Response: Thank you very much for your correction. We apologize for the unclear description of the legends of the figures, and we have re-annotated all the legends of the figures.

Question 4: Current form of Figure 6 is not suitable for publication in academic journal, a visualized heatmap might be better for reading.

Response: Thank you very much for your suggestion. Figure 6 has been replaced with a visual heatmap.

Question 5: A strongly suggested experiment for the supplement of the manuscript should be performed: a simple qRT-PCR of these *ABCC* genes during different stages of pigment deposition is not enough to elucidate their possible functions involved in proanthocyanidin accumulation, therefor, two parts of the experiment are advised to be added, 1) the phenotypes of brown-fibers in different stages of pigment deposition, 2) the content detection of proanthocyanidin in different brown-fiber developmental stages.

Response: Thank you very much for your suggestions. We have added two parts of the experiment. The detailed description of the supplementary experiments is the section of results in the revised manuscript about the Accumulation of PAs and Expression Analysis of *ABCC* Genes in Brown Cotton.

Question 6: The cis-element analysis of promoters of the *GhABCC* genes could be supplemented, to better understand the possible regulatory mechanism.

Response: Thank you very much for your comment. Through bioinformatics analysis, we speculate that *GhABCC27* may be related to the transport of proanthocyanidins, so we analyzed the cis-acting elements of the promoter of this gene. The detailed data are shown in Table 3.

Question 7: The Discussion section should be totally modified because of the narrow descriptions. Noticing that the discussion should be rewrote tightly focusing the obtained results in the manuscript.

Response: Thank you very much for your suggestion. We have rewritten the discussion section according to the reviewer’s suggestion.

Question 8: Line 202, the reason to select the materials of 6 DPA, 12 DPA, 18 DPA, 24 DPA, and 30 DPA fibers should be explained.

Response: Thank you very much for your suggestion. According to the reference [36], we found that the content of proanthocyanidins in brown cotton fibers changed significantly during these five periods, so we selected these periods to determine the relative expression of ABCC.

Question 9: Table 3 could be listed as a supplementary file.

Response: Thank you very much for your suggestion. We have listed Table 3 as a supplementary file.

Question 10: Line 233, the description of the sentence “A total of 178 *ABCC* genes were identified” is not accurate, actually, the manuscript identified 42 *ABCC* genes from *G. hirsutum*.

Response: Thank you very much for your correction. We apologize for the inaccuracy of this sentence, and we have completed the correction of this sentence.

Question 11: Other minor revisions

1)For the text descriptions, there exists amounts of short sentences in the manuscript, such as Lines 50-54, and of same words and phrases in different sentences such as “at present”, etc. Thus, the text descriptions should be carefully improved throughout the manuscript.

Response: Thank you very much for your comments. The text description in the manuscript has been improved.

2)Lines 62-64, the sentence “Therefor, ……” could be deleted.

Response: Thank you very much for your suggestion. Lines 62-64, the sentence “Therefor, ……” has been deleted.

3)Line 68, of the sentence “and they......”, the word “they” could be deleted.

Response: Thank you very much for your suggestion. Line 68, of the sentence “and they......”, the word “they” has been deleted.

4)Lines 88-89, more reports of *ABCC* genes in plants should be cited.

Response: Thank you very much for your comment. We have cited more reports about *ABCC* genes in plants.

5)Line 110, the words “in China” could be replaced by “worldwide”.

Response: Thank you very much for your suggestion. Line 110, the words “in China” has been replaced with “worldwide”.

6)Line 111, the word “cultivated” could be modified as “planted cultivar”.

Response: Thank you very much for your suggestion. Line 111, the word “cultivated” has been modified to “planted cultivar”.

7)Line 112, the reference [30] could be replaced by a newly published article of “Huang G, Wu Z, Percy RG, Bai M, Li Y, Frelichowski JE, Hu J, Wang K, Yu JZ, Zhu Y. Genome sequence of *Gossypium herbaceum* and genome updates of *Gossypium arboreum* and *Gossypium hirsutum* provide insights into cotton A-genome evolution. *Nat Genet*. 2020 May;52(5):516-524” for citation.

Response: Thank you very much for your correction. We apologize for the negligence in citing references. Reference [30] has cited the newly published article.

8)Line 128, the words “were very different” could be modified as “appeared significant differences”.

Response: Thank you very much for your suggestion. Line 128, the words “were very different” has been modified to “appeared significant differences”.

9)Line 133, the words “play different biological roles” could be modified as “potentially play different biological roles”.

Response: Thank you very much for your comment. Line 133, the words “play different biological roles” has been modified into “potentially play different biological roles”.

10)Line 140, a symbol between the word IV and The lost.

Response: Thank you very much for your comment. Line 140, a symbol between the word IV and The lost has been modified.

11)Line 152, the sentence “indicating that their functions may be similar” could be modified as “indicating the possibility of their similarity of functions”.

Response: Thank you very much for your suggestion. Line 152, the sentence “indicating that their functions may be similar” has been modified into “indicating the possibility of their similarity of functions”.

Responds to the reviewer’s comments:

Reviewer #2:

Question 1: The quality of figures needs improvement.

Response: Thank you very much for your comment. We have improved the quality of all figures.

Question 2. Some of the statements are awkward, such as “The resulting amino acid sequence…” in Line 273.

Response: Thank you very much for your corrections. We have revised the sentences in the article to make the expression as clear and accurate as possible.

Question 3. I suggest put the table 4 as supporting information.

Response: Thank you very much for your suggestion. We are sorry that there is no table 4 in the manuscript.

Question 4. Please removing AJE Completed files from the supporting information.

Response: Thank you very much for your suggestion. We have removed AJE Complete files from the supporting information.

Question 5. The reference for upland cotton genome you used is not incorrect.

Response: Thank you very much for your correction. We have cited the correct reference for upland cotton genome.

Question 6. In line 44, “one or several of these genes were selected for cloning and to verify their biological functions,..” This description is inaccurate, please modify it.

Response: Thank you very much for your correction. We have revised this part of the manuscript.

Once again, thank you very much for your comments and suggestions.
